# Medical occupational check-ups during the COVID-19 pandemic in the European Union

**DOI:** 10.1093/eurpub/ckae103

**Published:** 2024-06-18

**Authors:** Matyáš Fošum, Ladislav Štěpánek, Kateřina Ivanová, Marie Nakládalová

**Affiliations:** Department of Occupational Medicine, University Hospital Olomouc and Faculty of Medicine and Dentistry, Palacký University Olomouc, Olomouc, Czech Republic; Department of Public Health Protection, Ministry of Health of the Czech Republic, Prague, Czech Republic; Department of Occupational Medicine, University Hospital Olomouc and Faculty of Medicine and Dentistry, Palacký University Olomouc, Olomouc, Czech Republic; Department of Public Health, Faculty of Medicine and Dentistry, Palacký University Olomouc, Olomouc, Czech Republic; Department of Public Health, Faculty of Medicine and Dentistry, Palacký University Olomouc, Olomouc, Czech Republic; Department of Occupational Medicine, University Hospital Olomouc and Faculty of Medicine and Dentistry, Palacký University Olomouc, Olomouc, Czech Republic

## Abstract

Given the enormous scale of the COVID-19 pandemic affecting the healthcare sector, limited human resource capacity, and efforts to prevent the spread of COVID-19, occupational health protection could not escape changes. The aim was to identify and compare the regulations regarding the provision of medical occupational check-ups (MOCs) during the pandemic in all European Union member states (EU MS). The study employed the Delphi method, involving experts from EU MS to assess MOC regulations between January 2020 and May 2021. Experts were queried regarding the existence and specifics of MOC regulations, particularly for entrance and periodic MOCs at hazardous and non-hazardous workplaces. Out of the 27 EU MS surveyed, 13 EU MS did not regulate MOCs, while 14 EU MS (51.6%) regulated the provision of MOCs. The regulations were changes in the way MOCs were provided, modifications (postponement in time, alternative provision, e.g. using telemedicine or online connection, or replacing the medical certificate of fitness to work based on the MOC with a declaration by the worker), or interruption without compensation, even for hazardous works. The regulations were in effect for different lengths of time and varied in some countries during the study period. The cumulative duration of MOC interruptions in all EU MS during the study period was 137 months (7.5% of the cumulative study period of 1836 months). Given the different approaches to the provision of MOCs in EU MS, it has proved appropriate to develop an optimal unified framework plan for future similar situations.

## Introduction

At the end of 2019, local authorities in the city of Wuhan, Hubei Province, China, reported cases of an unknown type of pneumonia [1]. Subsequently, the disease was confirmed to be caused by severe acute respiratory syndrome coronavirus 2, the eighth known human coronavirus spread by droplets and person-to-person contact, which was key initiating epidemic control measures [2–5]. On 30 January 2020, the World Health Organization (WHO) designated the spread of coronavirus disease 2019 (COVID-19) as a public health emergency of international concern, or an extraordinary event which is determined to constitute a public health risk to other countries through the international spread of disease and to potentially require a coordinated international response [6]. On 11 March 2020, the WHO declared the outbreak of COVID-19 to be a pandemic. This negatively affected daily life and posed challenges to many long-established systems. The pandemic placed significant demands on healthcare systems worldwide, involving unprecedented pressure on intensive care capacity, laboratory testing, epidemiological surveillance, vaccination, etc. Given the enormous scale of the pandemic affecting the healthcare sector, limited human resource capacity and efforts to prevent the spread of COVID-19, occupational health protection (OHP), as part of public or global health, could not escape the changes [7–10]. In the European Union member states (EU MS), including the Czech Republic, preventive measures were ordered during the COVID-19 pandemic to protect global health, which also affected the work environment. In an effort to reduce contacts between sick and healthy people and to provide more effective healthcare to COVID-19 patients, occupational health services, including medical occupational check-ups (MOCs), justified by historical experience, were often sidelined. MOCs are an integral part of OHP and are enshrined in EU legislation, international conventions, and national laws [10–13].

MOCs are used to assess fitness for work and the impact of exposure to occupational risk factors on the health of employees. They serve to prevent work-related health problems and help the employer create a healthy workplace for the benefit of the physical and mental health of workers and their social environment, including the work team [12]. The WHO emphasized the importance of protecting workers and minimizing the impact on their health during the pandemic and promoted improved measures related to OHP, hygiene practices, and work organization [14]. Some EU MS are members of the International Labour Organization (ILO) and have ratified its international Occupational Health Services Convention No. 161 (C161). These countries are required to establish health surveillance of workers commensurate with the hazards of the work, requiring that the work be appropriate to the physical and mental health of the worker [15]. The ILO urged its member states to uphold and implement the ratified conventions and recommendations even during the epidemic crisis and to maintain and promote the three pillars of protection against the consequences of the COVID-19 pandemic on the labor market. The first pillar is the protection of workers in the workplace and the promotion of OHP; the second pillar is the support of employment and income; and the third pillar is the stimulation of the economy and demand [16]. During the COVID-19 epidemic, the ILO added a fourth pillar to the existing ones, the use of social dialogue between governments, workers, and employers to find solutions [17].

The study aimed to determine what measures in the system of providing MOCs were taken during the COVID-19 pandemic by the governments or other responsible authorities in the EU MS and whether, apart from the basic epidemic control action, they respected occupational health and safety, specifically the monitoring of long-term fitness for work in the form of MOCs. A secondary objective was to compare the approaches of the EU MS with regard to the ratification of C161. Our intention is to provide information that can inform the development of OHP planning procedures in times of crisis management and thus contribute to preparedness for possible future crises, including viral respiratory disease pandemics.

## Methods

The start of the study period was set at January 2020, corresponding to the reported outbreak of COVID-19 [18]. The study period ended in May 2021, when the pandemic peaked in Southeast Asia, the Americas, and Europe and when most epidemic control measures had been implemented [19]. In total, the study period lasted 17 months.

**Figure 1.**
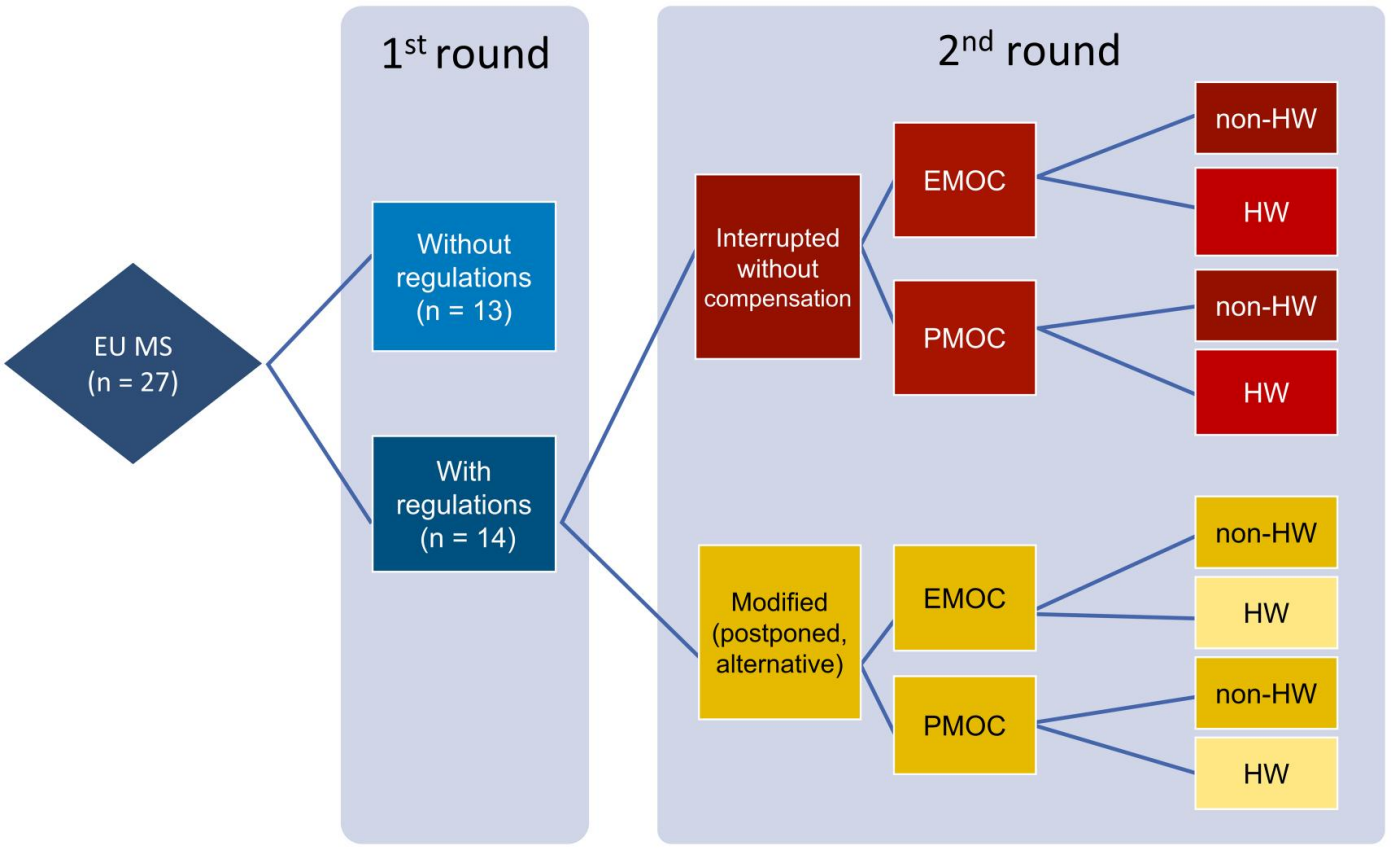
Diagram showing steps of the Delphi method. EU MS: European Union member states; EMOC: entrance medical occupational check-up; PMOC: periodic medical occupational check-up; HW: hazardous work.

From June to September 2021, data were collected with the help of the Permanent Representation of the Czech Republic to the EU, a diplomatic and political body connecting national authorities and EU institutions. Within this geographical and social framework, the EU MS (*n* = 27) were selected as narrators. The research design, based on the Delphi method [20], consisted of two rounds (Fig. 1). In the first round, experts involved in epidemic control measures in the EU MS at that time were approached through the permanent representations of all EU MS. The experts responded to the question of whether their countries had taken measures in the context of the COVID-19 pandemic that constituted a change in the system of MOC provision, or whether the provision of MOCs had been regulated in any way (e.g. provided in an alternative way or discontinued, i.e. not provided without compensation). After the first round, the EU MS were divided into two groups, the first comprising countries that had adopted regulations during the study period and the second consisting of countries that had not adopted any regulations.

After analyzing the responses from the first round, our OHP expert contacted the experts from the first group, that is, the countries adopting regulations. In a guided interview, the answer to the question asked in the first round was verified by the interviewer and, for the regulations identified, it was determined whether they were a modification of the MOCs or whether they were a form of discontinuation of the MOCs, that is, interruption without compensation (Table 1). At this stage, the MOCs were divided into four groups: entrance (EMOCs) or periodic (PMOCs) and those performed for hazardous works (HW) or non-hazardous works (non-HW).

**Table 1.** Questions asked to experts of European Union countries

First round	A question to experts of all European Union countries (*n* = 27)		Answer	
	Have measures been adopted in your country constituting a change in the system of MOC provision from January 2022 to May 2021?		Yes	No
Second round	Questions to experts who answered YES in the first round (*n* = 14)		Answer	
			Yes	No
	Were any of the MOCs interrupted entirely?	EMOC HW		
		EMOC non-HW		
		PMOC HW		
		PMOC non-HW		
	Were any of the MOCs provided modified or postponed?	EMOC HW		
		EMOC non-HW		
		PMOC HW		
		PMOC non-HW		
	If YES, indicate the period in which the individual measures were applied.			

EMOC HW: entrance medical occupational check-up for hazardous works; EMOC non-HW: entrance medical occupational check-up for non-hazardous works; PMOC HW: periodic medical occupational check-up for hazardous works; PMOC non-HW: periodic medical occupational check-up for non-hazardous works.

For all changes, their duration was ascertained. To compare the total duration of the regulations in the EU MS, the cumulative duration of the possible changes was calculated as the product of three components, namely, the number of all EU MS (*n* = 27), the number of months in the study period (*n* = 17), and the number of MOC types (*n* = 4), at 1836 months.

The indicators used to compare the data were time factor, type of MOCs, and type of MOC regulation. The common feature for all EU MS was the procedure for assessing medical fitness according to Article 14 of Directive 89/391/EEC, stating that measures shall be introduced in accordance with national law to ensure that workers receive health surveillance. The approaches of those EU MS that are members of the ILO and have ratified C161 (*n* = 12; BE, BG, HR, CZ, FI, DE, HU, LU, PL, SK, SI, SE) were compared with those adopted by the other EU MS [15].

The implemented epidemic control measures in the area of MOC provision in the EU MS were plotted on a Gantt chart. To compare the C161 ratifiers with the others, a chi-square test was used; the level of statistical significance was set at 0.05.

## Results

**Figure 2.**
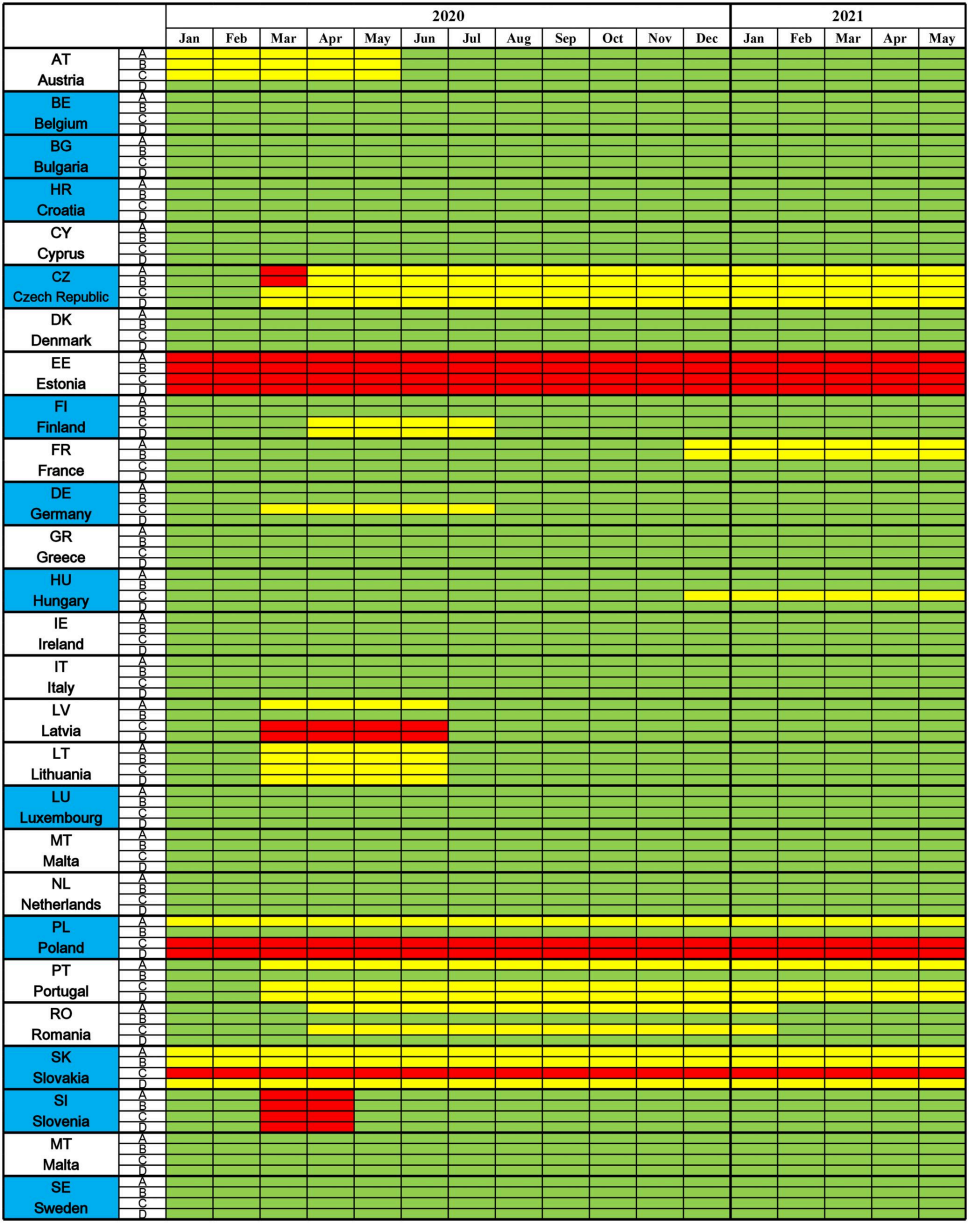
Gantt chart presenting regulation of medical occupational check-up provision in the European Union member states during the study period. Blue color: C161 ratifiers. A: entrance medical occupational check-ups for non-hazardous works; B: entrance medical occupational check-ups for hazardous works; C: periodic medical occupational check-ups for non-hazardous works; D: periodic medical occupational check-ups for hazardous works. Green color: no regulation; yellow color: modification (postponed, alternative); red color: interruption. The entire spreadsheet represents the cumulative study period (1836 months).

During the study period, 14 out of the 27 surveyed EU MS regulated the provision of MOCs (51.6%), whereas 13 EU MS introduced no regulations in this area (Fig. 2). Regulations were introduced in AT, CZ, EE, FI, FR, DE, HU, LV, LT, PL, PT, RO, SK, and SI (see Fig. 2 for country abbreviations). Regulations took different forms in different countries, such as government regulations (CZ, LV), laws passed by parliaments (AT, FR, PL, SK), or methodological guidance issued by competent authorities (FI). The Gantt chart (Fig. 2) shows for which time periods and types of MOCs the regulations were in place.

Regulation in the form of interruptions of all types of MOC (entrance/periodic for hazardous/non-hazardous works) was in place in Estonia throughout the study period. Slovenia also adopted the same regulation, but only for two months at the beginning of the period (March–April 2020). The Czech Republic allowed the interruption of entrance MOCs without compensation for one month (March 2020). Poland suspended all periodic MOCs for the entire period, as did Slovakia, but only for non-hazardous works. Latvia interrupted all periodic MOCs for four months, from March to June 2020. Overall, six EU MS (CZ, EE, LV, PL, SK, and SI) decided to discontinue MOCs, albeit certain types and for a limited time.

Regulations aimed at modifying MOCs were more frequent than those leading to a complete interruption and were implemented by 12 EU MS (AT, CZ, FI, FR, DE, HU, LV, LT, PL, PT, RO, and SK). Modifications most often consisted of

- postponement in time through extending the validity of existing and expiring medical certificates of fitness to work,
- replacing the MOC and the certificate with a declaration by the employee or applicant,
- conducting the health assessment remotely, e.g. using telemedicine or online connection,
- having the MOC done by a healthcare provider other than the incumbent.

The MOC modification was in effect for the longest time in Slovakia, lasting for the entire study period and covering all types of MOCs except periodic MOCs for non-hazardous works, which were interrupted without compensation. The option of modifying MOCs was also relatively extensive in the Czech Republic. In Poland, it was possible to modify entrance MOCs for non-hazardous works throughout the period. Extensive modifications were also possible in Portugal from March 2020 until the end of the study period for all MOCs. The other countries allowed modifications of some types of MOCs for shorter time periods (AT, FI, FR, DE, HU, LV, RO).

The cumulative duration of MOC interruptions in all EU MS during the study period was 137 months, or 7.5% of the cumulative study period of 1836 months (EMOC HW 20 months, EMOC non-HW 20 months, PMOC HW 40 months, and PMOC non-HW 57 months). The cumulative duration of MOC modifications was 257 months, or 14% of the cumulative study period (EMOC non-HW 92 months, EMOC HW 46 months, PMOC non-HW 64 months, and PMOC HW 55 months).

### Comparison of the approaches of EU MS ratifying and not ratifying ILO C161

There was no statistically significant difference between the two groups in the number of countries with MOC regulations, whether they were modifications or interruptions (Table 2). However, there were differences in the duration of regulations between the two groups of countries. On average, the cumulative duration of MOC regulations was longer in ILO members and C161 ratifiers. There was a significant difference in the duration of modifications between the groups, with C161 ratifiers having considerably longer measures, especially regarding entrance MOCs for hazardous works. On average, C161 ratifiers interrupted entrance MOCs for both hazardous and non-hazardous works for a statistically significantly shorter time than the other countries.

**Table 2.** Comparison of the types and duration of medical occupational check-up (MOC) regulations in European Union member states (EU MS) regarding the ratification of the international Occupational Health Services Convention No. 161 (C161)

MOC regulations	EU MS (*n* = 27)	C161 ratifiers (*n* = 12)	C161 non-ratifiers (*n* = 15)	*P* value (C161 ratifiers vs. non-ratifiers)
No. of countries regulating MOCs (modifications + interruptions)	14	7	7	0.735
**Of which:**				
MOC interruptions	6	4	2	0.325
MOC modifications	12	6	6	0.919
**Cumulative duration of MOC regulations (months)**				
Cumulative duration of all regulations	394	206	188	0.005
**MOC interruptions**	**137**	**61**	**76**	**0.985**
**Of which:**				
EMOC non-HW interruptions	20	3	17	0.008
EMOC HW interruptions	20	3	17	0.008
PMOC non-HW interruptions	57	36	21	0.005
PMOC HW interruptions	40	19	21	0.700
**MOC modifications**	**257**	**145**	**112**	**< 0.001**
**Of which:**				
EMOC non-HW modifications	92	48	44	0.146
EMOC HW modifications	46	31	15	0.002
PMOC non-HW modifications	64	30	34	0.701
PMOC HW modifications	55	36	19	0.008

EMOC HW: entrance medical occupational check-up for hazardous works; EMOC non-HW: entrance medical occupational check-up for non-hazardous works; PMOC HW: periodic medical occupational check-up for hazardous works; PMOC non-HW: periodic medical occupational check-up for non-hazardous works.

## Discussion

The impact of the COVID-19 pandemic on the work environment was studied by Ranka et al., who reported changes in the behavior of UK occupational health services to cope with the pandemic [8]. Similar conclusions were also reached in other studies [9, 21]. Thus, the question is whether addressing this emergency situation respected, apart from the basic objective of epidemic control measures, other systems that protect public health and global health aspects such as occupational health and safety including MOCs [22].

The present study shows that 13 EU MS did not regulate MOCs, while the other 14 (AT, CZ, EE, FI, FR, DE, HU, LV, LT, PL, PT, RO, SK, and SI) regulated different types of MOCs by implementing a range of measures valid for different periods of time. Guided interviews with responsible experts from each country revealed that in the first months of the study period, MOC rules were adapted in Slovenia for two months, in Lithuania, Latvia, and Finland for four months, and in Austria and Germany for five months. Throughout the entire 17-month period, MOCs were regulated by three EU MS, namely, Estonia, Poland, and Slovakia; besides modifications, these countries opted, to varying degrees, for interrupting MOCs without compensation.

Thus, the present study confirmed that in most EU MS, the OHP systems diverged from normal due to the implementation of general epidemic control measures that prioritized the protection of living conditions over established OHP, including MOCs, during the COVID-19 pandemic. Commenting on the situation, B. O. Iddins, Medical Director of the Oak Ridge National Laboratory Health Services Division, states that the major tasks of pandemic planning must reinforce the objective to “prevent the spread of the infection or contagion” and that this includes avoiding the pitfall of “optimism bias” [23].

On the other hand, in a number of EU countries, MOCs were not regulated at all, were modified only for a short period of time, or were performed in at least a limited, alternative form. According to Ranka et al., 90% of UK occupational health services continued their activities, including MOCs, during the COVID-19 pandemic. MOC providers responded promptly to the situation by, for example, offering out-of-hours services or employing extra staff [8]. The authors emphasize the need of occupational health services, including MOCs, to be prepared for crisis situations [8]. Similarly, Journeay and Burnstein reported on the situation during the H5N1 influenza epidemic and concluded that although occupational health and safety services were provided during the epidemic, pandemic planning for occupational health workers in the case of respiratory illness is inadequate [24].

Entry MOCs for hazardous works, where failure to respect medical contraindications poses a health risk not only to the worker but also, in some cases, to their surroundings, were interrupted on average for a statistically significantly shorter period of time in C161 ratifiers. In contrast, modified, or alternative, MOCs, especially for hazardous works, were allowed to continue for statistically significantly longer periods in these countries. This may indicate that these countries paid more attention to the provision of occupational health services, especially those related to hazardous works. However, the experience of the EU MS in their implementation on the ground would contribute to an overall assessment of the impact of measures regulating MOCs.

Our guided interviews with experts also discussed whether there is a functional setup of common unified processes for the protecting living and working conditions during emergencies and whether there is a functional communication network for these situations. Unfortunately, at the time of the study, there was no unified plan or communication network applicable to OHP and, in particular, to the provision of MOCs. This can be considered, among other things, as a consequence of the fact that EU legislation on public health protection is not harmonized with national legislations [25, 26]. Almost two decades ago, Smith et al. called for the establishment of procedures and a communication network for emergencies as a precautionary measure. They documented that within the pharmaceutical and biotechnology industry, 40 out of 50 companies surveyed did not have a plan for emergencies [27]. According to Iddins, public health guidance should be clear, authoritative, evidence-based, effectively communicated, and thoroughly coordinated when multiple stakeholders are involved. All potential problems and barriers must be resolved before the actual threat occurs [23].

The good news is that in September 2021, the Health Emergency Preparedness and Response Authority, a new Directorate-General within the European Commission, was established [28, 29]. The need for a functional communication network within the EU and the implementation of common procedures for pandemics is also reflected in the European Parliament resolution of 12 July 2023 on the COVID-19 pandemic: lessons learned and recommendations for the future, or 2022/2076(INI) [29]. An important element in the EU’s preparedness for future threats is Regulation (EU) 2022/2371 of the European Parliament and of the Council on serious cross-border threats to health and repealing Decision No 1082/2013/EU. Each EU MS in coordination with the European Commission should develop its national preparedness and response plan. The Czech Republic is already working on such a plan. Preparations for public health emergencies also include the proposed International Treaty on Pandemic Prevention, Preparedness, and Response.

Occupational health and safety is a flexible, responsive, and adaptable field. However, the diversity of national approaches, confirmed in addition to our study by the European Parliament’s final report on the COVID-19 pandemic, also brings the possibility of a new perception of the necessity of the whole institution of MOCs and fitness for work assessment [26]. However, the interruption of entrance MOCs for hazardous works was possible (with the exception of Estonia, which interrupted all MOCs throughout the study period) only at the beginning of the period and only for one month in the Czech Republic and two months in Slovenia, suggesting the importance of these MOCs even during the pandemic.

The ILO and WHO emphasized protecting workers and minimizing the impact on their health, with prevention being identified as the first priority [30]. ILO and WHO documents promoted improved OHP policies, hygiene practices, and forms of work organization [14, 31–33]. ILO member states were guided by recommendations that promote and emphasize the importance of preventive healthcare [30]. The Committee of Experts on the Application of Conventions and Recommendations systematically promoted ILO conventions in the area of occupational health and safety [34]. It specifically mentions that occupational health services play a key role in monitoring workers’ health and providing guidance for adapting workplace practices and procedures and developing safety protocols, as stipulated in C161 [30].

The ILO and WHO recommendations for better occupational health and safety were endorsed by, among others, the Japanese Trade Union Confederation, or RENGO [35]. At its Global Summit entitled COVID-19 and the World of Work, the ILO, supported by all its members, called for global support for worker risk management against the threats posed by the COVID-19 epidemic [16]. Employers should update their risk assessments and measures to reduce the level of new COVID-19 risks [36].

In conclusion, the approach of EU MS to the regulation of occupational health services during the first year and a half of the COVID-19 pandemic was inconsistent, with regulations ranging from alternative MOCs to postponement to complete interruption. Similarly, the duration of the regulations varied considerably among EU MS. As many as 48% of the countries did not regulate MOCs at all. The European framework for a global approach to health encourages member states, through the WHO, the ILO, and the European Agency for Safety and Health at Work, to make continuous and sustainable occupational health and safety a cornerstone of public and global health. It is therefore unacceptable to neglect occupational safety and health in favor of the seemingly superior principle of protecting public and global health. The results of the present study point indirectly to the lack of a common emergency plan for the provision of MOCs as one of the areas of occupational health and safety. There is a need for transparent occupational health and safety emergency measures to be implemented in a coordinated way in the EU, in dialogue with the economic and social partners. The present paper suggests a potential communication network for such a procedure.

## Data Availability

The data underlying this article will be shared on reasonable request to the corresponding author. Key points Occupational check-ups, as an integral part of occupational health protection, are enshrined in European Union legislation, international conventions, and national laws. To reduce contact between sick and healthy people and to provide more effective healthcare to COVID-19 patients, medical occupational check-ups were often sidelined. The approach of European states to the regulation of occupational health services during the pandemic was inconsistent, with regulations ranging from alternative check-ups to postponement to complete interruption. There is a lack of a common emergency plan for the provision of occupational check-ups as one of the areas of occupational health and safety.
